# Circulating miR‐19a‐3p and miR‐19b‐3p characterize the human aging process and their isomiRs associate with healthy status at extreme ages

**DOI:** 10.1111/acel.13409

**Published:** 2021-06-23

**Authors:** Cristina Morsiani, Lucia Terlecki‐Zaniewicz, Susanna Skalicky, Maria Giulia Bacalini, Salvatore Collura, Maria Conte, Federica Sevini, Paolo Garagnani, Stefano Salvioli, Matthias Hackl, Johannes Grillari, Claudio Franceschi, Miriam Capri

**Affiliations:** ^1^ DIMES‐Department of Experimental, Diagnostic and Specialty Medicine University of Bologna Bologna Italy; ^2^ Christian Doppler Laboratory for Biotechnology of Skin Aging Vienna Austria; ^3^ Department of Biotechnology Institute of Molecular Biotechnology BOKU – University of Natural Resources and Life Sciences Vienna Austria; ^4^ TAmiRNA GmbH Vienna Austria; ^5^ IRCCS Istituto delle Scienze Neurologiche di Bologna Bologna Italy; ^6^ Interdepartmental Center "Alma Mater Research Institute on Global Challenges and Climate Change (Alma Climate)" University of Bologna Bologna Italy; ^7^ Applied Biomedical Research Center (CRBA) S. Orsola‐Malpighi Polyclinic Bologna Italy; ^8^ CNR Institute of Molecular Genetics "Luigi Luca Cavalli‐Sforza" – Unit of Bologna Bologna Italy; ^9^ Department of Laboratory Medicine Clinical Chemistry Karolinska Institutet Karolinska University Hospital Stockholm Sweden; ^10^ Austrian Cluster for Tissue Regeneration Vienna Austria; ^11^ Ludwig Boltzmann Institute for Experimental and Clinical Traumatology Vienna Austria; ^12^ Laboratory of Systems Medicine of Healthy Aging and Department of Applied Mathematics Lobachevsky University Nizhny Novgorod Russia

**Keywords:** aging, extreme phenotypes, isomiRs, longevity, microRNAs, miR‐19a/b‐3p

## Abstract

Blood circulating microRNAs (c‐miRs) are potential biomarkers to trace aging and longevity trajectories to identify molecular targets for anti‐aging therapies. Based on a cross‐sectional study, a discovery phase was performed on 12 donors divided into four groups: young, old, healthy, and unhealthy centenarians. The identification of healthy and unhealthy phenotype was based on cognitive performance and capabilities to perform daily activities. Small RNA sequencing identified 79 differentially expressed c‐miRs when comparing young, old, healthy centenarians, and unhealthy centenarians. Two miRs, that is, miR‐19a‐3p and miR‐19b‐3p, were found increased at old age but decreased at extreme age, as confirmed by RT‐qPCR in 49 donors of validation phase. The significant decrease of those miR levels in healthy compared to unhealthy centenarians appears to be due to the presence of isomiRs, not detectable with RT‐qPCR, but only with a high‐resolution technique such as deep sequencing. Bioinformatically, three main common targets of miR‐19a/b‐3p were identified, that is, SMAD4, PTEN, and BCL2L11, converging into the FoxO signaling pathway, known to have a significant role in aging mechanisms. For the first time, this study shows the age‐related increase of plasma miR‐19a/b‐3p in old subjects but a decrease in centenarians. This decrease is more pronounced in healthy centenarians and was confirmed by the modified pattern of isomiRs comparing healthy and unhealthy centenarians. Thus, our study paves the way for functional studies using c‐miRs and isomiRs as additional parameter to track the onset of aging and age‐related diseases using new potential biomarkers.

## Graphical abstract

Small RNA deep sequencing on plasma‐derived RNA from 12 donors (young and old subjects, healthy, and unhealthy centenarians) was performed: 79 differentially expressed c‐miRs were identified. MiR‐19a/b‐3p increased at old age but decreased at extreme age, as confirmed by RT‐qPCR in 49 subjects. The decrease was more relevant in healthy centenarians, also confirmed by the modified pattern of isomiRs. Three common targets of miR‐19a/b‐3p were identified converging into the FoxO signaling pathway.

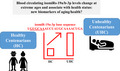

## 1 INTRODUCTION

Demographic projections report a worldwide increase in human life expectancy over the last few decades. The age‐related stratification of population demography emphasizes the critical importance of identifying new strategies able to counteract or slowdown aging process with the aim of combating the onset of almost all age‐related diseases and geriatric syndromes (ARD‐GS), as suggested by Geroscience (Kennedy et al., 2014). In this perspective, a thorough comprehension of molecular and cellular aspects underlying human aging is of primary importance to improve quality of life and to promote healthy longevity according with WHO (https://www.who.int/ageing/decade‐of‐healthy‐ageing).

As highlighted by our team, aging is not only the major risk factor for ARD‐GS, but it shares the same molecular mechanisms with the above mentioned diseases, as a sort of continuum (Collura et al., 2020; Franceschi et al., 2018; Morsiani et al., 2019). Thus, the study of underpinning mechanisms would favor the discovery of molecular targets for both anti‐aging therapies and for associated pathologies.

In order to follow this strategy, we here chose to study circulating microRNAs (c‐miRs) and their targets as a basis for understanding molecular mechanisms of aging in centenarians. They are an extraordinary model for the study of healthy aging and longevity, since centenarians largely avoid or postpone major age‐related diseases and are characterized by decelerated aging (Giuliani et al., 2017). Moreover, we propose an improved model, considering healthy and unhealthy centenarians with the aim of grasping the differences in terms of blood circulating molecules, as previously published (Teo et al., 2019). Here, the same cross‐sectional study was performed analyzing the four groups composed of young, old, and two cohorts of centenarians divided into healthy and unhealthy individuals. Healthy centenarians have both optimal cognitive and physical status, while unhealthy are in opposite extreme conditions.

This new model allows us to reveal the spreading of aging signals in the blood (Franceschi et al., 2017), including c‐miRs (Olivieri et al., 2017). MiRs are a highly conserved class of small non‐coding RNAs (18–25 nt) and are key regulators of gene expression at post‐transcriptional level. C‐miRs have been shown to impact on various biological processes, such as proliferation, differentiation, and apoptosis. Furthermore, they are recognized as mediators of “inflammaging,” a term used to describe the age‐associated chronic inflammation, and they can be considered as important members of the senescence‐associated secretory phenotype (SASP) (Franceschi et al., 2017; Olivieri et al., 2013a; Terlecki‐Zaniewicz et al., 2018). In fact, some c‐miRs are aberrantly regulated upon aging and during ARD‐GS (Fehlmann et al., 2020; Hackl et al., 2017; Heilmeier et al., 2016; Jung & Suh, 2014; Olivieri et al., 2013b); thus, they are powerful epigenetic biomarkers to trace aging/ARD‐GS and longevity, as recently suggested by our team (Olivieri et al., 2017).

The current study aimed to investigate the differential abundance of c‐miRs in non‐fractionated plasma samples derived from different aged groups by small RNA‐seq and RT‐qPCR in order to identify potential/novel biomarker and regulators of gene expression.

## 2 RESULTS

### 2.1 Quality control and normalization of deep sequencing data

The profiling of small RNAs was performed on plasma samples of 12 individuals with different ages and health conditions: 3 young donors, 3 old donors, 3 healthy, and 3 unhealthy centenarians. Summaries of total reads obtained from sequencing and reads mapped on human genome are reported in Table S1 and Figure S1, respectively. Expression counts were defined as Tags Per Million (TPM), and after normalization, 420 miRs were found as commonly expressed in all samples. About 1000 miRs with TPM ≥1 and about 300 with TPM ≥20 were identified as shown in Figure S2. For subsequent analyses, miRs with TPM ≥20 were used.

### 2.2 Discovery phase: c‐miRs by small RNA‐seq in aging and longevity

**FIGURE 1:**
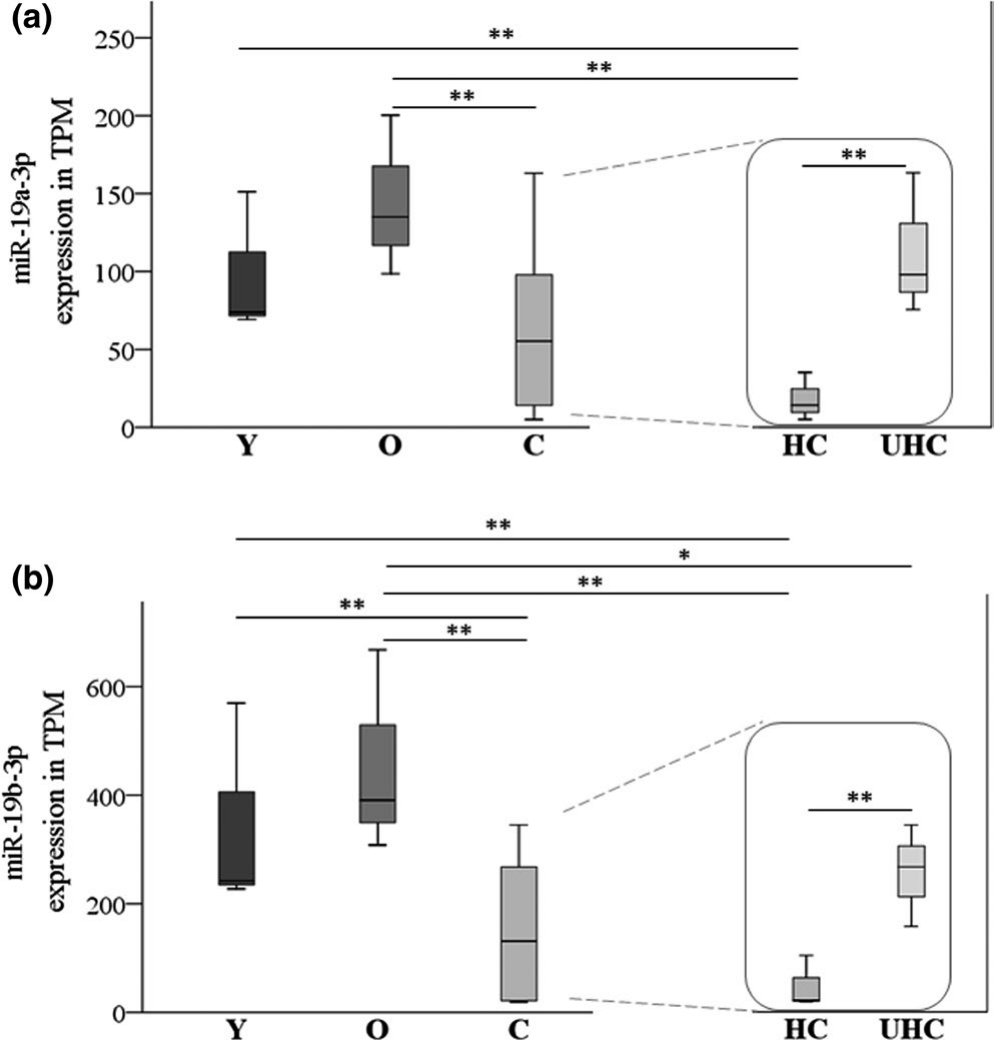
Expression of miR‐19a/b‐3p by small RNA‐seq. Expression level is reported as TPM counts for miR‐19a‐3p (panel a) and miR‐19b‐3p (panel b). Data are shown as box plots (with median) for each group, that is, 3 young donors (Y), 3 old donors (O), and 6 centenarians (C). Centenarians are also analyzed according to their health status, that is, 3 healthy (HC) and 3 unhealthy centenarians (UHC) as reported on the right. Statistical analysis was assessed applying the glmQLFTest implemented by EdgeR statistical software package in R v3.6.3. In this analysis, both *p* value thresholds were considered and indicated as follows: **<0.01; *<0.05. Particularly, in panel a for miR‐19a‐3p: Y vs. HC *p* = 4.79E‐05; O vs. C *p *= 0.0035; O vs. HC *p* = 1.16E‐06; HC vs. UHC *p *= 0.0001. In panel b for miR‐19b‐3p: Y vs. C *p *= 0.0021; Y vs. HC *p *= 4.79E‐07; O vs. C *p* = 6.29E‐05; O vs. HC *p* = 1.84E‐08; O vs. UHC *p *= 0.045; HC vs. UHC *p *= 7.06E‐05

In order to identify age‐associated changes, miR abundance analysis was performed according to a classical cross‐sectional study design (young “Y” *vs* old “O” individuals *vs* centenarians “C”) for discovery phase. In addition, we compared healthy and unhealthy centenarians (“HC” vs. “UHC”) to identify miRs associated with health status at extreme ages. Applying small RNA‐seq, we obtained 79 miRs differentially expressed among “Y,” “O,” “HC,” and “UHC” groups. The results are shown in Table 1, and TPM values for each group are reported in Table S2. MiR‐19a‐3p and miR‐19b‐3p turned out to be the most highly significant miRs identified comparing healthy and unhealthy centenarians, also having a significant modification through the different age groups. The sequencing data are shown in Figure 1. The miR‐19 family comprises miR‐19a and miR‐19b where the mature miRs differ solely in a single base; thus, we will further use the annotation miR‐19a/b‐3p. Those two miRs were further selected for the validation phase by RT‐qPCR using samples obtained from 49 donors.

**TABLE 1:** Seventy‐nine significant miRs comparing the four age groups

miRNA	Young versus Old		Young versus Healthy Centenarians		Young versus Unhealthy Centenarians		Old versus Healthy Centenarians		Old versus Unhealthy Centenarians		Healthy versus Unhealthy Centenarians	
	logFC	*p* Value	logFC	*p* Value	logFC	*p* Value	logFC	*p* Value	logFC	*p* Value	logFC	*p* Value
**hsa‐miR‐19b‐3p**	−0.45	0.44195	3.22	0.00000	0.74	0.20705	3.67	0.00000	1.19	0.04460	−2.47	0.00007
**hsa‐miR‐19a‐3p**	−0.61	0.32607	2.72	0.00005	0.12	0.85258	3.32	0.00000	0.72	0.24727	−2.60	0.00010
hsa‐miR‐4433b‐3p	−0.62	0.33335	0.12	0.85214	−2.10	0.00143	0.73	0.25130	−1.49	0.02113	−2.22	0.00083
hsa‐miR‐145‐5p	−0.36	0.53771	1.06	0.07674	−0.87	0.14561	1.42	0.01820	−0.50	0.39550	−1.93	0.00168
hsa‐miR‐10b‐5p	−0.23	0.67868	0.98	0.08227	−0.78	0.16622	1.21	0.03251	−0.55	0.32911	−1.76	0.00225
hsa‐miR‐6503‐3p	0.04	0.96595	3.80	0.01057	−0.76	0.50871	3.76	0.01061	−0.80	0.47532	−4.56	0.00318
hsa‐miR‐4485‐3p	−2.65	0.00097	−4.27	3.88E−07	−2.08	0.01359	−1.62	0.02325	0.57	0.44840	2.19	0.00447
hsa‐miR‐887‐3p	−0.63	0.45000	1.36	0.15532	−1.37	0.12395	2.00	0.03631	−0.74	0.39679	−2.73	0.00636
hsa‐miR‐598‐3p	0.48	0.48107	−0.10	0.88196	1.98	0.00975	−0.58	0.39502	1.50	0.04816	2.08	0.00679
hsa‐miR‐296‐5p	−0.04	0.96455	1.85	0.04817	−0.80	0.35595	1.88	0.04359	−0.77	0.37654	−2.65	0.00705
hsa‐miR‐2277‐5p	1.12	0.34804	−1.25	0.30078	5.31	0.05158	−2.37	0.04967	4.19	0.14532	6.56	0.00969
hsa‐miR‐6842‐5p	2.54	0.04601	−0.89	0.44412	5.51	0.03971	−3.43	0.00821	2.97	0.33053	6.40	0.01190
hsa‐miR‐4739	2.19	0.22166	2.19	0.37088	−3.96	0.05106	0.00	1.00000	−6.14	0.00233	−6.14	0.01199
hsa‐miR‐122‐5p	−1.02	0.07037	0.14	0.80639	1.52	0.00788	1.15	0.04072	2.53	0.00002	1.38	0.01525
hsa‐miR‐31‐5p	−1.88	0.02831	0.97	0.29556	6.71	0.00185	2.84	0.00225	8.58	0.00001	5.74	0.01571
hsa‐miR‐409‐3p	0.98	0.08592	−0.61	0.28229	0.74	0.19500	−1.59	0.00602	−0.24	0.66902	1.35	0.01930
hsa‐miR‐3688‐3p	0.51	0.57958	1.91	0.06064	7.49	0.00049	1.40	0.16211	6.98	0.00155	5.58	0.02543
hsa‐miR‐4741	1.47	0.28966	−2.17	0.21178	−5.49	0.00111	−3.64	0.03514	−6.96	0.00008	−3.32	0.02750
hsa‐miR‐197‐5p	−1.78	0.07627	−0.91	0.38254	−3.09	0.00359	0.87	0.36925	−1.31	0.17164	−2.18	0.03232
hsa‐miR‐4656	0.00	1.00000	0.00	1.00000	−5.38	0.01415	0.00	1.00000	−5.38	0.00918	−5.38	0.03401
hsa‐miR‐539‐5p	0.88	0.23262	−1.40	0.05622	0.23	0.76622	−2.28	0.00249	−0.65	0.40096	1.63	0.03479
hsa‐miR‐4669	−1.18	0.23694	0.47	0.66589	5.92	0.01592	1.65	0.12971	7.10	0.00176	5.45	0.03707
hsa‐miR‐487b‐3p	0.81	0.20689	−1.03	0.10723	0.29	0.65768	−1.84	0.00490	−0.52	0.41977	1.32	0.04349
hsa‐miR‐32‐5p	1.18	0.07970	1.40	0.04207	2.92	0.00014	0.22	0.74798	1.73	0.02074	1.52	0.04563
hsa‐miR‐3168	−1.61	0.08382	−1.52	0.11232	−3.32	0.00070	0.09	0.91904	−1.71	0.05189	−1.80	0.04747
hsa‐miR‐127‐3p	0.67	0.23946	−0.86	0.13218	0.27	0.63184	−1.53	0.00829	−0.40	0.48431	1.13	0.04883
hsa‐miR‐3200‐3p	−0.54	0.42913	0.74	0.28573	2.30	0.00370	1.28	0.06604	2.84	0.00036	1.56	0.05016
hsa‐miR‐1226‐5p	0.00	1.00000	−2.41	0.20171	−6.16	0.00363	−2.41	0.17430	−6.16	0.00200	−3.75	0.05919
hsa‐miR‐431‐5p	0.88	0.15574	−0.83	0.17949	0.32	0.60413	−1.72	0.00671	−0.56	0.37086	1.16	0.06627
hsa‐miR‐3176	0.39	0.64896	0.98	0.27615	3.28	0.00720	0.59	0.50588	2.89	0.01766	2.30	0.06791
hsa‐miR‐96‐5p	0.10	0.90504	3.08	0.00478	0.93	0.34204	2.98	0.00600	0.82	0.39365	−2.15	0.07055
hsa‐miR‐323b‐3p	1.04	0.09867	−0.96	0.12592	0.17	0.78606	−2.00	0.00186	−0.87	0.16990	1.13	0.07450
hsa‐miR‐134‐5p	0.46	0.42993	−1.59	0.00792	−0.54	0.36132	−2.05	0.00072	−1.00	0.09112	1.05	0.07512
hsa‐miR‐6817‐3p	2.34	0.14746	−0.81	0.65359	−3.91	0.02720	−3.16	0.07749	−6.26	0.00112	−3.10	0.07989
hsa‐miR‐3675‐5p	−1.79	0.24878	−5.27	0.00953	0.00	1.00000	−3.48	0.04572	1.79	0.54421	5.27	0.08191
hsa‐miR‐101‐5p	−0.26	0.71269	1.98	0.00902	3.70	0.00011	2.24	0.00323	3.96	0.00003	1.72	0.08236
hsa‐miR‐215‐5p	−0.69	0.32213	1.32	0.07211	0.00	0.99959	2.01	0.00655	0.69	0.34299	−1.32	0.08442
hsa‐miR‐6087	−0.82	0.20554	0.94	0.15914	−0.21	0.75576	1.76	0.00870	0.62	0.34695	−1.14	0.09072
hsa‐miR‐379‐5p	0.37	0.54712	−1.79	0.00436	−0.78	0.20433	−2.16	0.00067	−1.15	0.06316	1.01	0.10252
hsa‐miR‐186‐3p	−0.27	0.70727	2.04	0.01009	0.69	0.37896	2.31	0.00344	0.96	0.21567	−1.35	0.10372
hsa‐miR‐144‐3p	−0.21	0.70470	0.53	0.33695	1.43	0.01089	0.74	0.18196	1.64	0.00372	0.90	0.10491
hsa‐miR‐362‐5p	−0.38	0.71589	1.64	0.17067	6.14	0.01375	2.02	0.08769	6.52	0.00701	4.50	0.11413
hsa‐miR‐382‐5p	0.20	0.73529	−1.71	0.00398	−0.79	0.17501	−1.91	0.00141	−0.99	0.09144	0.92	0.11452
hsa‐miR‐93‐5p	0.21	0.70737	0.74	0.17907	1.58	0.00522	0.54	0.33068	1.37	0.01468	0.83	0.13379
hsa‐miR‐576‐5p	0.58	0.41817	1.19	0.10990	2.42	0.00401	0.61	0.40736	1.84	0.02733	1.23	0.14897
hsa‐miR‐654‐5p	0.05	0.93377	−1.79	0.00711	−0.85	0.19904	−1.85	0.00531	−0.90	0.16897	0.94	0.14940
hsa‐miR‐186‐5p	−0.15	0.78205	1.58	0.00543	0.79	0.16078	1.74	0.00239	0.94	0.09432	−0.80	0.15387
hsa‐miR‐193b‐3p	−0.46	0.65282	2.39	0.05210	6.46	0.00723	2.84	0.01896	6.91	0.00284	4.07	0.16243
hsa‐miR‐493‐5p	0.55	0.36327	−1.21	0.04985	−0.40	0.51433	−1.76	0.00472	−0.95	0.12077	0.81	0.18626
hsa‐miR‐183‐5p	−0.13	0.82428	1.53	0.00798	0.79	0.16261	1.66	0.00423	0.92	0.10662	−0.74	0.19450
hsa‐miR‐9‐5p	0.80	0.25104	−0.69	0.32167	−1.57	0.02605	−1.49	0.03314	−2.37	0.00092	−0.88	0.20336
hsa‐miR‐4532	−0.35	0.55131	1.75	0.00339	1.08	0.06821	2.10	0.00052	1.42	0.01669	−0.68	0.24933
hsa‐miR‐124‐3p	1.71	0.25311	−0.63	0.67105	−2.34	0.11166	−2.34	0.13531	−4.05	0.01000	−1.71	0.24997
hsa‐miR‐20b‐5p	0.36	0.56185	1.43	0.02227	2.16	0.00093	1.08	0.08313	1.81	0.00518	0.73	0.25272
hsa‐miR‐144‐5p	0.64	0.29544	1.60	0.00999	2.31	0.00035	0.97	0.11459	1.67	0.00825	0.71	0.25844
hsa‐miR‐18b‐5p	0.09	0.91441	2.57	0.00663	1.39	0.15476	2.49	0.00836	1.30	0.18066	−1.19	0.27704
hsa‐miR‐29c‐3p	−0.32	0.59801	1.45	0.02253	0.77	0.22160	1.77	0.00555	1.09	0.08311	−0.68	0.28407
hsa‐miR‐1908‐3p	−1.32	0.16448	−2.21	0.02439	−3.11	0.00195	−0.88	0.31677	−1.78	0.04769	−0.90	0.31923
hsa‐miR‐16‐5p	0.81	0.13559	2.23	0.00008	1.73	0.00192	1.41	0.01047	0.92	0.09373	−0.50	0.35897
hsa‐miR‐3135b	−1.52	0.08229	−2.56	0.00473	−1.79	0.05810	−1.04	0.21765	−0.27	0.76138	0.77	0.39688
hsa‐miR‐1299	2.64	0.00043	2.40	0.00149	3.07	0.00029	−0.24	0.74745	0.43	0.61108	0.66	0.43638
hsa‐miR‐432‐5p	0.37	0.51302	−1.24	0.03080	−0.83	0.14741	−1.61	0.00545	−1.20	0.03706	0.41	0.46475
hsa‐miR‐4488	−1.27	0.05904	−2.53	0.00028	−2.05	0.00300	−1.26	0.05394	−0.79	0.23068	0.48	0.46720
hsa‐miR‐550a‐3p	0.34	0.60175	1.76	0.00992	2.23	0.00189	1.42	0.03573	1.89	0.00792	0.47	0.51269
hsa‐miR‐106b‐5p	0.12	0.83321	1.84	0.00226	2.20	0.00033	1.72	0.00422	2.08	0.00066	0.37	0.53731
hsa‐miR‐15b‐5p	0.77	0.20034	1.33	0.02926	1.69	0.00661	0.56	0.35178	0.92	0.13351	0.35	0.56058
hsa‐miR‐194‐5p	0.46	0.48101	1.65	0.01576	2.04	0.00437	1.18	0.07923	1.58	0.02592	0.40	0.57754
hsa‐miR‐4466	−1.86	0.03462	−2.64	0.00372	−3.10	0.00092	−0.78	0.33855	−1.24	0.13824	−0.46	0.58416
hsa‐miR‐323a‐3p	0.73	0.24902	−1.17	0.06692	−0.86	0.18003	−1.90	0.00343	−1.59	0.01425	0.31	0.62297
hsa‐miR‐5189‐3p	−4.27	0.00756	−2.05	0.23291	−2.84	0.13676	2.22	0.12249	1.43	0.35910	−0.79	0.65950
hsa‐miR‐490‐5p	−0.89	0.33800	−2.97	0.00172	−2.61	0.00769	−2.09	0.01553	−1.72	0.05496	0.37	0.67376
hsa‐miR‐3138	−0.77	0.28981	−1.74	0.01899	−2.00	0.00831	−0.97	0.17370	−1.23	0.09157	−0.26	0.72111
hsa‐miR‐192‐5p	−0.27	0.62776	1.41	0.01426	1.27	0.02673	1.68	0.00368	1.54	0.00750	−0.14	0.80729
hsa‐miR‐185‐5p	−0.35	0.53115	1.56	0.00621	1.65	0.00391	1.91	0.00092	2.00	0.00055	0.09	0.86978
hsa‐miR‐3200‐5p	0.18	0.79454	2.51	0.00123	2.63	0.00161	2.33	0.00249	2.45	0.00314	0.12	0.88698
hsa‐miR‐15a‐5p	0.65	0.24229	1.46	0.01017	1.53	0.00692	0.80	0.14993	0.88	0.11489	0.08	0.88829
hsa‐miR‐451a	0.58	0.28405	2.56	0.00001	2.61	0.00001	1.98	0.00043	2.03	0.00030	0.06	0.91812
hsa‐miR‐3161	2.24	0.09828	5.79	0.00668	5.79	0.03406	3.55	0.11453	3.55	0.25406	0.00	1.00000
hsa‐miR‐217	−5.31	0.00585	0.00	1.00000	0.00	1.00000	5.31	0.01905	5.31	0.06827	0.00	1.00000

### 2.3 Validation phase of small RNA‐seq results by RT‐qPCR

MiR‐19a/b‐3p sequencing data were validated by RT‐qPCR in plasma samples obtained from 49 different aged donors, that is, 16 young donors, 16 old donors, 11 healthy centenarians, and 6 unhealthy centenarians, as detailed in methods section.

**FIGURE 2:**
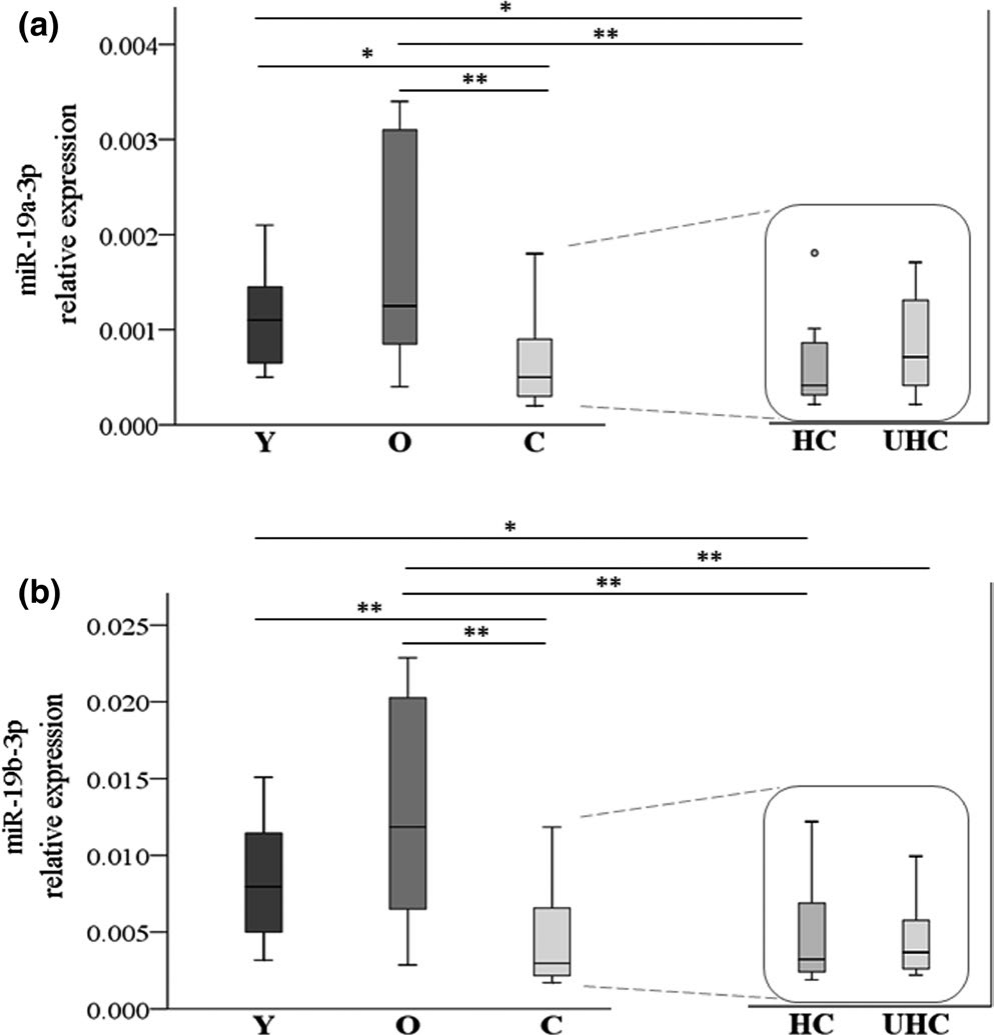
Validation of miR‐19a/b‐3p by RT‐qPCR. RT‐qPCR results of miR‐19a‐3p (panel a) and miR‐19b‐3p (panel b) are shown as box plots (with median) of relative expression for each group, that is, 16 young donors (Y), 16 old donors (O), and 17 centenarians (C). Centenarians are also analyzed according to their health status, that is, 11 healthy (HC) and 6 unhealthy centenarians (UHC) as reported on the right. Statistical analysis was assessed by nonparametric Kruskal–Wallis test, and significant p values are indicated as follows: **<0.01; *<0.05. Particularly, in panel a for miR‐19a‐3p: Y vs. C *p *= 0.032; O vs. C *p *= 0.001; Y vs. HC *p *= 0.029; O vs. HC *p *= 0.001. In panel b for miR‐19b‐3p: Y vs. C *p *= 0.009; Y vs. O *p *< 0.0001; Y vs. HC *p *= 0.020; O vs. HC *p *= 0.001; O vs. UHC *p *= 0.005

RT‐qPCR validation results are reported in Figure 2 (panels a and b), showing miR‐19a/b‐3p data in all age groups. In addition, healthy and unhealthy centenarians were compared. Validation analysis confirmed data obtained with small RNA‐seq along with the age groups. MiR‐19a/b‐3p increases in old group and decreases in centenarians. However, the different levels of both miRs in healthy versus unhealthy centenarians were not confirmed by qPCR.

**FIGURE 3:**
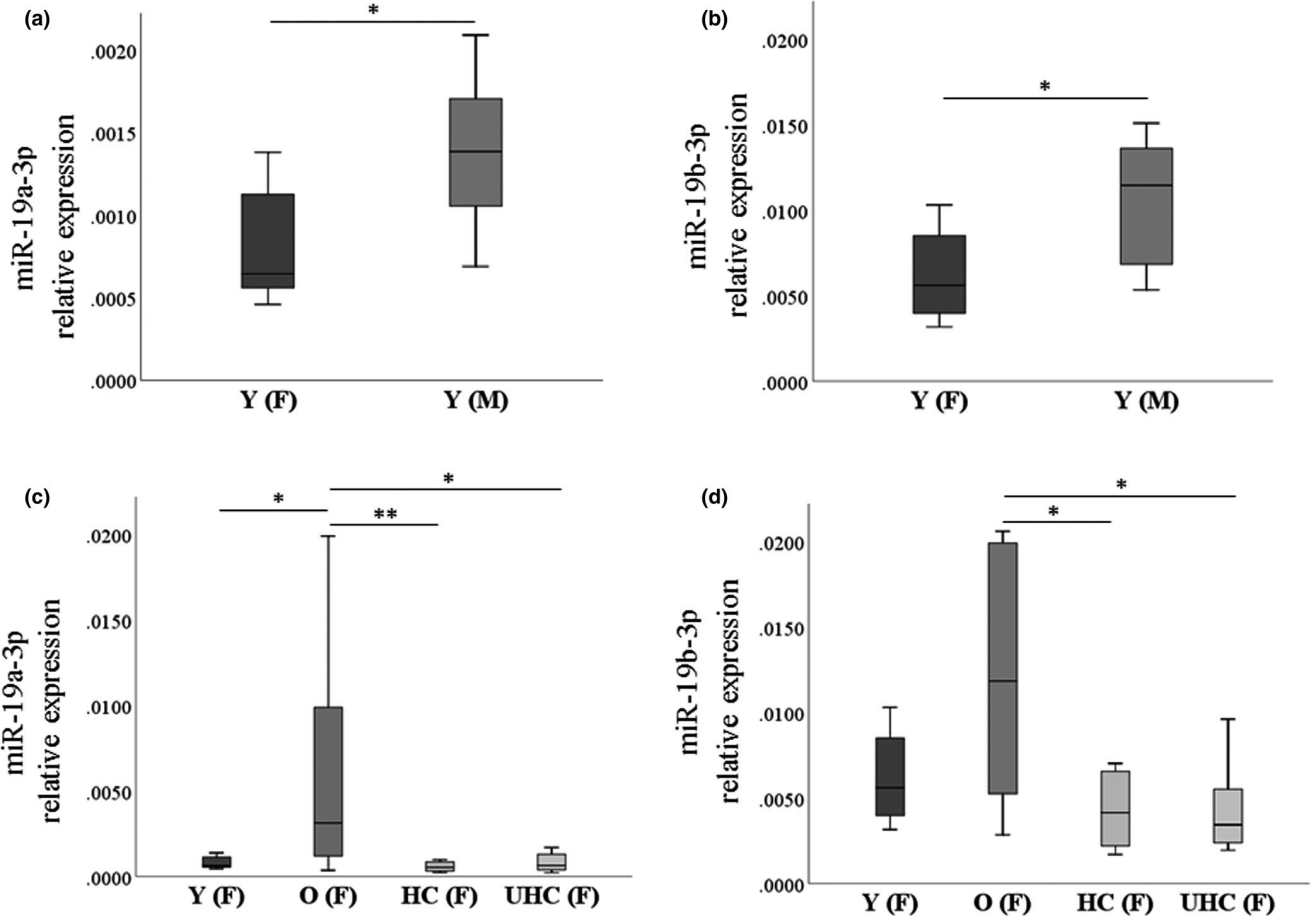
miR‐19a/b‐3p analyzed according to gender in the validation phase cohort. RT‐qPCR data were analyzed in young subjects according to gender, dividing in 8 young female Y (F) and 8 young male Y (M), for miR‐19a‐3p (panel a) and miR‐19b‐3p (panel b). Analysis for female counterpart was applied among 8 young women Y (F), 8 old women O (F), 8 healthy women centenarians HC (F), and 6 unhealthy women centenarians UHC (F). Data are shown as box plots (with median) of relative expression for each group. Statistical analysis was assessed by nonparametric Mann–Whitney and Kruskal–Wallis test. Significant *p* values are indicated as follows: **<0.01; *<0.05. Particularly, in panel a for miR‐19a‐3p *p *= 0.015 and in panel b for miR‐19b‐3p *p *= 0.028. In panel c for miR‐19a‐3p Y vs. O *p *= 0.050; O vs. HC *p *= 0.002; O vs. UHC *p *= 0.032. In panel d for miR‐19b‐3p: O vs. HC *p *= 0.015; O vs. UHC *p *= 0.020

Validation data were also analyzed according to sex within the groups when the number of male and females was balanced. In the young group, a significant difference between male and females was found in both miR‐19a/b‐3p, as reported in Figure 3 (panels a and b), being the expression higher in males. Further analysis was performed in all groups considering only females being the number of subjects comparable. The results are shown in Figure 3 (panels c and d), and this stratification highlighted the increase in old compared to young females, showing a greater similarity between young and centenarian females.

RT‐qPCR miR‐19a/b‐3p levels were correlated with hematobiochemical parameters in all centenarians, and the matrix correlation is reported in Figure S3. Seven significant correlations were found as follows: miR‐19a‐3p negatively correlates with creatinine (*p* = 0.02), while miR‐19b‐3p negatively correlates with RDW‐CV (*p* = 0.053), with eosinophils (*p* = 0.032), with creatinine (*p* = 0.046) and miR‐19b‐3p positively correlates with WBC (*p* = 0.044), with neutrophils (*p* = 0.02), and with GPT (*p* = 0.031). Comparing healthy and unhealthy centenarians, only a few hematobiochemical parameters resulted significant (mean corpuscolar hemoglobin concentration, albumin, and total protein *p* < 0.05) as shown in Table S4.

### 2.4 IsomiRs contribution in aging and longevity

**FIGURE 4:**
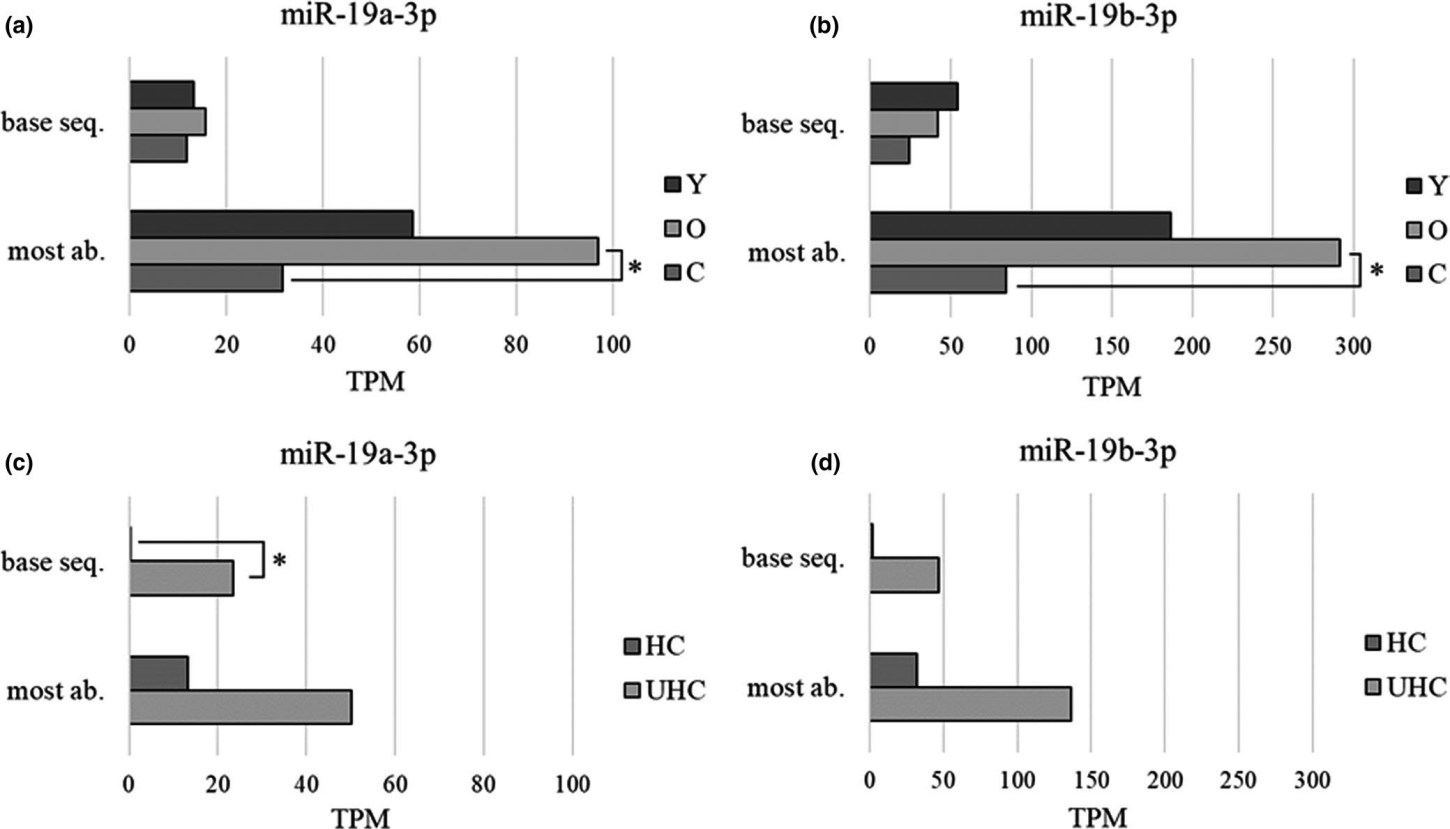
IsomiRs of miR‐19a‐3p and miR‐19b‐3p in aging and longevity. The base and the most abundant sequences of miR‐19a‐3p (panels a and c) and of miR‐19b‐3p (panels b and d), obtained by small RNA‐seq, are shown as TPM mean value for each group. Panels a and b show data from 3 young donors (Y), 3 old donors (O), and 6 centenarians (C). Panels c and d show centenarians according to their health status, that is, 3 healthy (HC) and 3 unhealthy centenarians (UHC). Statistical analysis was assessed by wilcox.test function implemented in R; *p* < 0.05 (*)

The contribution of isomiRs, that is, the variations of the base sequence annotated in miRBase, in the sequencing results were evaluated. Sequencing data show that the most abundant isoform of miR‐19a/b‐3p in plasma is a truncated form at 3′ (iso_3p) lacking the last two nucleotides (Figure S4). Both the annotated base sequences in miRBase and the most abundant sequences of miR‐19a‐3p and miR‐19b‐3p, reported as TPM mean value for each group, are shown in Figure 4. The most abundant sequence of both miRs is significantly decreased in centenarians compared with old donors (panels a and b). Centenarians’ group was further analyzed according to health status; results show an increase of the miRBase sequence in unhealthy compared to healthy centenarians (panels c and d), but only isomiR‐19a‐3p differs significantly.

### 2.5 Pathway analysis

**FIGURE 5:**
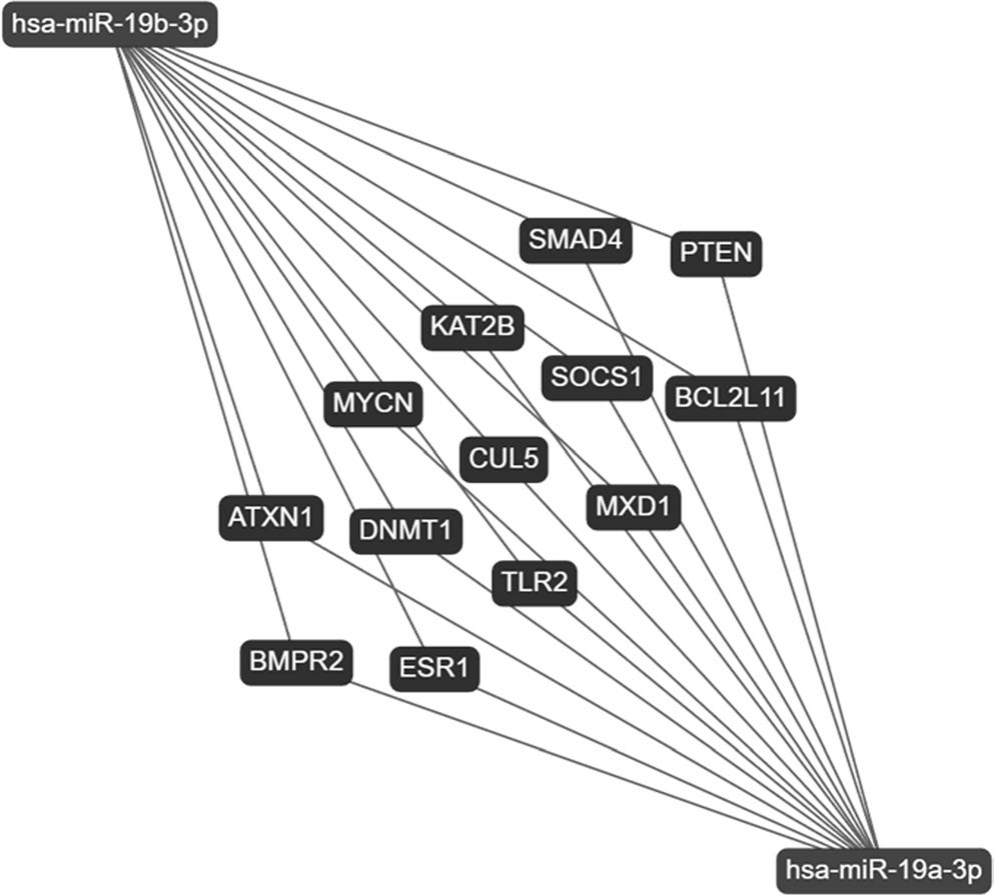
Target network analysis of miR‐19a/b‐3p. Common validated targets of miR‐19a‐3p and miR‐19b‐3p obtained with experimental strong evidence. Target network interactions have been determined via miRTargetLink

Target gene network analysis of the differentially expressed miRs was determined via miRTargetLink (https://ccb‐web.cs.uni‐saarland.de/mirtargetlink/), and shared molecular targets between miR‐19a‐3p and miR‐19b‐3p with experimentally validated publications were raised up. In particular, the thirteen most significant common targets of both miRs have been found, as reported in Figure 5.

KEGG analysis via mirPath (http://snf‐515788.vm.okeanos.grnet.gr/) was performed considering all validated targets of miR‐19a‐3p and miR‐19b‐3p. Using pathway intersection testing, 28 pathways turned out to be significantly regulated. The most significant pathway (*p* < 10^−325^) is related to proteoglycans in cancer, while at 5th position the FoxO signaling pathway was found (*p* = 3.19 × 10^−07^). This pathway contains the highest number of shared targets, that is, SMAD4, PTEN, and BCL2L11, among those identified by miRTargetLink analysis. Table S5 shows the list of all the 28 pathways with the related level of significance.

## 3 DISCUSSION

The work has been performed on plasma c‐miRs by means of deep sequencing technique using a discovery design based on a cross‐sectional study with differently aged individuals. A cohort of 3 donors for each group, that is, young, old, healthy centenarians, and unhealthy centenarians, was investigated. A subsequent validation phase was conducted by RT‐qPCR in plasma samples obtained from 16 young donors, 16 old donors, 11 healthy centenarians, and 6 unhealthy centenarians. This up‐graded model, which takes into account two extreme and very rare phenotypes of centenarians, was recently proposed by our team (Teo et al., 2019). In this respect, healthy centenarians have both optimal cognitive (SMMSE 24–30) and physical status (ADL = 5), while unhealthy are at the opposite condition (bedridden and not able to reply to the interview). Surprisingly, hematobiochemical parameters did not greatly differ between the two groups. In particular, mean corpuscolar hemoglobin concentration, albumin, and total protein resulted decreased in unhealthy compared to healthy centenarians, probably due to anemia and organ disfunction more pronounced in unhealthy status.

Sequencing data analysis was performed comparing young, old, healthy, and unhealthy centenarians, and 79 c‐miRs were found differentially expressed. Among these, there are several miRs with a known role in aging/ARD‐GS. An example is miR‐31, found increased in plasma microvesicles of elderly and patients with osteoporosis, possibly involved in the pathogenesis of age‐related and impaired bone formation (Weilner et al., 2016) and identified as markers of aging in human liver (Capri et al., 2017). In particular, the comparison between healthy and unhealthy centenarians revealed 11 significantly changed miRs, and 8 out of 11 resulted increased in unhealthy centenarians, thus suggesting a general trend of increased miRs upon unhealthy conditions.

Notably, the most significantly different miRs are miR‐19a‐3p and miR‐19b‐3p. These miRs belong to the miR‐17/92 cluster that maps to human chromosome 13 and encodes for six individual miRs, (i.e., miR‐17, miR‐18a, miR‐19a, miR‐20a, miR‐19b‐1, and miR‐92a) (Mendell, 2008). The organization and sequences are highly conserved among vertebrates, and the human genome contains two paralogues, the miR‐106b/25 and the miR‐106a/363 cluster. In addition to miR‐19a/b‐3p, sequencing analysis revealed miR‐106a/b‐5p, miR‐18b‐5p, and miR‐20b‐5p, belonging to these paralogues, significantly modified with age. In particular, miR‐19a‐3p and miR‐19b‐3p share the same seed region (Concepcion et al., 2012) and are involved in many processes such as cell cycle, proliferation, and apoptosis, having as main targets PTEN and BCL2L11 (Mogilyansky & Rigoutsos, 2013). Interestingly, PTEN is also a target of miR‐21, an inflamma‐miR found decreased in centenarians when compared with old group (Olivieri et al., 2012) as miR‐19a/b‐3p.

Currently, limited data are available on the age‐related changes of c‐miRs and/or at human extreme ages (Dluzen et al., 2017; Olivieri et al., 2017). Unifying conclusions are difficult due to the presence of several variables, such as biological sample, method, and normalization procedure. Some studies have already explored various components of peripheral blood for different analyses, such as the genome‐wide miR screening performed in whole blood of centenarians and nonagenarians *versus* younger individuals (ElSharawy et al., 2012); the miR‐profile obtained from B cells of individuals aged 50–90 and centenarians (Gombar et al., 2012); and the analysis of miR‐expressions achieved in mononuclear cells from centenarians, octogenarians, and young individuals (Serna et al., 2012). As far as plasma is concerned, only two studies explore c‐miRs. A recent paper showed a significant decrease of three miRs, that is, miR‐425‐5p, miR‐21, and miR‐212 in centenarians compared with controls aged from 30 to 50 years (Balzano et al., 2017). However, authors did not explore miRs level with a profiling technique, but a priori selection applying RT‐qPCR was performed. Olivieri et al. (2012) investigated miR‐profiling in individuals aged from 20 to 100 years and among the significantly increased miRs in octogenarians compared to centenarians, two resulted in common with our data, that is, miR‐19b‐3p and miR‐186‐5p. In this perspective, the current data extend the significant increase of miR‐19b‐3p and miR‐186‐5p also in the 70‐year‐old group compared to centenarians.

As far as miR‐19a‐3p is concerned, we previously showed an up‐regulation in the adipose tissue obtained by postmenopausal in comparison with premenopausal younger women (Kangas et al., 2017). We did not find changes of miR‐19a‐3p in the plasma, but the current results suggest an age‐related increase in the plasma detectable at older ages than previously investigated.

Serum miR‐19a/b‐3p were described to be significant discriminators of patients with idiopathic and postmenopausal osteoporotic low‐traumatic fractures (Kocijan et al., 2016), and miR‐19b‐3p was confirmed significantly up‐regulated in older women with osteoporotic vertebral fractures (Zarecki et al., 2020), showing their potential to be marker of age‐related conditions.

MiR‐19b‐3p was previously described as a pivotal player in human aging. MiR profiling of four different cell types in replicative senescence and three different types of *ex vivo* tissue was achieved (Hackl et al., 2010). Thereby, a common down‐regulation of miR‐17, miR‐19b, miR‐20a, and miR‐106a was found. The decrease in these miRs correlated with the increased transcript levels of cdk inhibitor p21/CDKN1A. In a recent study, the changes of circulating miRs after resistance exercise were investigated (Margolis et al., 2017). The analysis performed at the baseline of the study showed that serum miR expression profiles were significantly predictive of chronological aging through a stepwise discriminant analysis, based on miR‐19b‐3p, miR‐206, and miR‐486 able to correctly classify the participants by age. Serna et al. found downregulated miR‐19b‐3p in blood peripheral mononuclear cells from octogenarians in respect to both young donors and centenarians (Serna et al., 2012). It is reasonable that the level of intracellular expression of these miRs may be different from the level found in circulation, and it would be interesting to evaluate the expression of miRs and their targets in the various blood cells and cell tissue, even if quite complex in humans, aiming at deepening the contribution of expression and function of those miRs.

The impact of the age‐related changes of miR‐19a/b‐3p may be explored in their molecular targets. Figure 5 shows the most significant common targets of miR‐19a‐3p and miR‐19b‐3p, backed up by powerful experimental methods as resulted from the miRTargetLink analysis. All of them are involved in crucial processes, such as PTEN described as promoter of longevity (Ortega‐Molina & Serrano, 2013). SMAD4 is a mediator of TGF‐β signal transduction, a pathway important in senescence and aging (Tominaga & Suzuki, 2019). BCL2L11 belongs to the BCL‐2 protein family with apoptotic regulator function (Tower, 2015). SOCS1, known as suppressor of cytokine signaling, acts via the JAK/STAT pathway inhibiting JAK directly, and senescence‐associated secretory phenotype (SASP) can be suppressed by inhibiting the JAK pathway in senescent cells (Xu et al., 2015). Noteworthy, both miR‐19a/b‐3p and their targets, that is, SMAD4, PTEN, and BCL2L11, converge on FoxO signaling pathway and are also relevant for IGF‐mTOR (Hay, 2011), thus two well‐known pathways associated with aging and longevity.

MiR‐19a/b‐3p resulted differentially expressed when young donors were stratified according to sex, being increased in young male compared to female subjects. Analyzing all groups and considering only women, differences emerged highlighting the increase of miR‐19a‐3p in old compared to young subjects, suggesting a specific, inhibitory, and likely estrogen‐dependent effect in old women. In fact, miR‐19a/b‐3p are also common regulator of ESR1 and are in turn controlled by estrogen levels in a regulatory circuit with a negative feedback and is thus proposed to be sexually dimorphic miRs (Dai & Ahmed, 2014). MiR‐19b‐3p has a precursor in the miR‐106a/363 cluster located on the X chromosome, even if this cluster is normally expressed at lower levels (Mogilyansky & Rigoutsos, 2013) and highlights possible gender differences in human aging (Kangas et al., 2017; Matarrese et al., 2019). Thus, the unexpected decrease of miR‐19a/b‐3p at extreme ages could also be considered a biomarker associated with female longevity.

The validation phase, performed by RT‐qPCR, did not confirm the differences of miR‐19a/b‐3p between the healthy and unhealthy centenarians obtained by sequencing data. MiR‐19a/b‐3p resulted correlated with WBC, RDW‐CV, neutrophils, eosinophils, creatinine, and GPT in all centenarians, suggesting their possible involvement in the regulation of health status parameters and immune system.

To better disentangle the unexpected results obtained by RT‐qPCR, we investigated the presence of isomiRs‐19a/b‐3p in sequencing data. IsomiRs are not rare and were estimated to contribute to half of the miRs profile in human cells (Zhao, 2020). They are classified into 5′, 3′, or polymorphic (internal) isomiRs, depending on the site of variation, and can be derived from alternative Drosha or Dicer processing, RNA editing, or non‐templated nucleotide addition (Gebert & MacRae, 2019). IsomiRs also showed gender or race specific expression patterns (Guo et al., 2016), and some specific isoforms have been found useful in distinguishing different types of cancer representing potential biomarkers (Telonis et al., 2017; Zhao, 2020). However, up to date, the function and biological significance of the majority of these isomiRs remain unclear.

Our first evidence is that the two selected miRs, ‐19a/b‐3p and ‐19b‐3p, reveal a variety of isoforms, up to 38 and 76, respectively, and the most abundant sequence does not correspond to the current miRBase reference sequence, as already described from the first annotation of isomiRs (Morin et al., 2008). PCR based assays, microarrays, and northern blots do not normally discriminate among highly similar sequences, and they are constructed on the base sequence annotated on miRBase. As isomiRs are at least as prevalent as their canonical sequences or even more abundant, this suggest that miR detection studies published using these assays may have based their conclusions on partial data (Karlsen et al., 2019). Furthermore, this could be the reason why we were unable to validate small RNA‐seq results by RT‐qPCR comparing healthy and unhealthy centenarians. In fact, comparing healthy and unhealthy centenarians, results suggest the increase of the base sequence of miR‐19a‐3p in unhealthy condition and similarly, a trend is observed for miR‐19b‐3p. Moreover, the most abundant sequences of both miRs were significantly decreased in centenarians compared to old donors. These crucial findings open new questions and pave the way for a new perspective on the role and function of the different isomiRs‐19a/b‐3p on health conditions and aging. IsomiRs have not yet a recognized biological function, but early data from literature suggest an impact on gene expression regulation and a cytokine‐mediated increase of isomiR patterns (Haseeb et al., 2017). In this respect, specific 3’ isomiRs having a shorter length, as well as those identified here, were found not able to control molecular pathways as the base sequence does (Yu et al., 2017) and their expression was modulated by Interferon type I (Nejad et al., 2018). These findings may be considered as a general effect of 3′ short length isomiRs with a powerful potential for gene expression regulation, but further studies are needed to confirm this hypothesis.

One limitation of the current study is the lack of information on miRs‐19a/b‐3p expression level during the whole life of centenarians. The model used in this study was a cross‐sectional, being not feasible a longitudinal study until extreme human ages.

Overall, miR‐19a/b‐3p were found decreased at blood/systemic level in centenarians compared with old subjects, and a different pattern of isomiRs‐19a‐3p was associated with aging process and with health status at extreme age. In this perspective, the functional role of isomiRs on aging mechanisms deserves additional studies with the aim of identifying new levels of regulation, such as those abrogating miR inhibitory effects, as valuable key factors for aging and age‐related diseases onset.

## 4 EXPERIMENTAL PROCEDURES

### 4.1 Experimental design and study participants

Twelve donors were included in the discovery phase for the small RNA sequencing, divided in 4 groups of study as follows: 3 young donors with 25 year‐old ±0.5 (SD) (Y), 3 old donors with 71 year old ±1.6 (O), 3 healthy centenarians (HC), and 3 unhealthy centenarians (UHC) being 101.8 year old ±1.1. In all groups, the F:M ratio was 2:1, except for unhealthy centenarians that consisted of all females. All subjects included in the study were recruited in Bologna, the Ethical Committee of Sant’ Orsola‐Malpighi Hospital (Bologna, Italy) approved the protocol (EM/26/2014/U with reference to 22/2007/U/Tess). A detailed questionnaire describing their lifestyle, physical, and cognitive status was administrated to centenarians. The healthy centenarians were defined by good cognitive performance, that is, MMSE (Mini‐Mental State Examination), ability to walk and a high ADL (Activities of Daily Living) score. The unhealthy centenarians were demented without the possibility to perform the MMSE and bedridden. Young and old subjects are representative of the general population and realistic aging. Hematobiochemical parameters are reported in Table S3. These twelve subjects were previously investigated in terms of circulating cell‐free DNA by our team and described in details for health conditions and drugs therapies (Teo et al., 2019).

Validation phase was conducted on 49 volunteers, including those previously selected for discovery phase, divided as follows (average age ± standard deviation): (i) 16 young donors, 8 females and 8 males (30.1 ± 3.4); (ii) 16 old donors, 8 females and 8 males (70.8 ± 1.9); (iii) 17 centenarians, that is, 11 healthy centenarians, 8 females and 3 males, and 6 unhealthy centenarians, only females (101.4 ± 1.3).

Hematobiochemical parameters were collected from all subjects recruited for the validation phase (Table S3). Differences between healthy and unhealthy centenarians were considered (Table S4). Nonparametric Kruskal–Wallis and Mann–Whitney tests were applied using IBM SPSS Statistics, version 26 (IBM Corp., Armonk, NY, USA).

### 4.2 Sample processing for sequencing

Total RNA was isolated from 2 ml of plasma samples with the miRNeasy Mini kit (Qiagen), and the protocol was adapted for the extraction from plasma. As circulating RNA is present in very low amount, RNA quantification was not possible; therefore, exogenous controls, that is, UniSp2, UniSp4, and UniSp5 (RNA Spike‐In, Qiagen), were introduced at the beginning of RNA extraction and their recovery was used as indicator of RNA isolation quality.

### 4.3 cDNA library preparation

For cDNA library preparation, CleanTag Ligation Kit (TriLink BioTechnologies) was used. Each sample was associated with a different Index Primer (Primers #1‐12) from the Set 1 for Illumina technology (TriLink BioTechnologies) to run for the sequencing all 12 samples in one lane. A purification step after PCR reaction was performed using QIAquick PCR Purification Kit (Qiagen) to remove primers, nucleotides, enzymes, mineral oil, salts, and other impurities from DNA samples. cDNA libraries were quantified by Agilent DNA 1000 Kit using Agilent 2100 Bioanalyzer (Agilent). A gel purification step is required to run amplified cDNA library and select only miRs library according to the size, cutting the corresponding miR band (~130–140 bp). cDNA library was quantified again after the purification by Agilent DNA 1000 chip.

### 4.4 Sequencing data analysis

The sequencing was completed by Exiqon A/S Company on the NextSeq500 Illumina platform, using single‐end read and 50 nt as number of sequencing cycles.

Data preparation and analysis was based on Cap‐miRSeq pipeline (Sun et al., 2014) following these steps: (i) FASTQC and Cutadapt were applied to verify the quality of the data before and after the adapters’ trimming. The samples showed overall good data quality with the vast majority of the data obtained, presenting higher Q‐score than Q30, when a score of 30 equals an accuracy of 99.9% for the base‐calling (Cock et al., 2010); (ii) miRDeep2 was used to identify miR expression profiling where the sequencing reads were mapped to the reference human genome in miRBase v.21; (iii) normalization process was performed by Tags Per Million (TPM) method according to the total tag count in each sample. Data were filtered for count threshold, ≥20 TPM to achieve a robust validation using a different technique as RT‐qPCR; (iv) differential expression analysis was implemented by EdgeR statistical software package in R v3.6.3, applying the glmQLFTest and data were considered significant with a *p* value <0.01.

### 4.5 Validation and statistics

Total RNA was isolated from 100 μl of plasma using the Total RNA purification kit (Norgen Biotek Corporation). The protocol was modified adding 20 fmol of cel‐miR‐39 (Qiagen) at the lysis step as spike‐in control to monitor RNA isolation. RT‐qPCR was performed through TaqMan technologies (Thermo Fisher Scientific) following the manufacturer's protocol.

Data were normalized to cel‐mir‐39 measured in each sample, and relative expression was calculated with the delta Ct method. Nonparametric tests were used to perform statistic evaluation, using SPSS software, and *p* value < 0.05 was considered statistically significant.

Validation data were correlated with hematobiochemical parameters in centenarians by Spearman's correlation test performed with GraphPad Prism software v.9, and *p* value ≤0.05 was considered significant.

### 4.6 Pathway analysis

Identification of common target genes was determined via miRTargetLink, a tool for automating analysis on human miR‐mRNA interactions in the form of interaction networks (Hamberg et al., 2016). Targets with strong experimental evidence, like reporter gene assay, were selected and filtered only those in common between the two selected miRs. The identification of the pathways was determined via Kyoto encyclopedia of genes and genomes (KEGG) with *p* values < 0.05, using the mirPath software on DIANA tools (Vlachos et al., 2015).

### 4.7 IsomiRs analysis

On the selected miRs, isoform sequences and their abundances were investigated with IsomiR‐SEA v.1.6 (Urgese et al., 2016). This tool performs reads alignment based on miRs databases by considering the miRNA:mRNA interaction pairing aspects. Statistical analysis was performed using the wilcox.test function implemented in R (using default parameters and setting continuity correction to false) and considering a *p* value < 0.05.

## CONFLICT OF INTEREST

M.H. and J.G. are co‐founders of TAmiRNA GmbH. J.G. is co‐founder of Evercyte GmbH.

## AUTHOR CONTRIBUTIONS

C.M., L.TZ., and S. Sk. performed the experiments; C.M. and MG.B. completed the bioinformatical analysis; C.M., S.C., M.C., and F.S. elaborated data and drafted the manuscript; M.H., J.G., C.F., and M.C conceived and designed the study; L.TZ., P.G., S. Sa., M.H., J.G., C.F., and M.C. critically revised the manuscript.

## Supporting information

Fig S1

Fig S2

Fig S3

Fig S4

Supplementary Material

## Data Availability

The data that support the findings of this study are available from the corresponding author upon request.
