# Supplementary figures and images for: Circulating miR‐19a‐3p and miR‐19b‐3p characterize the human aging process and their isomiRs associate with healthy status at extreme ages
Source: Aging Cell. 2021 Jun 23;20(7):e13409. doi: 10.1111/acel.13409 (PMC8282272; doi:10.1111/acel.13409)

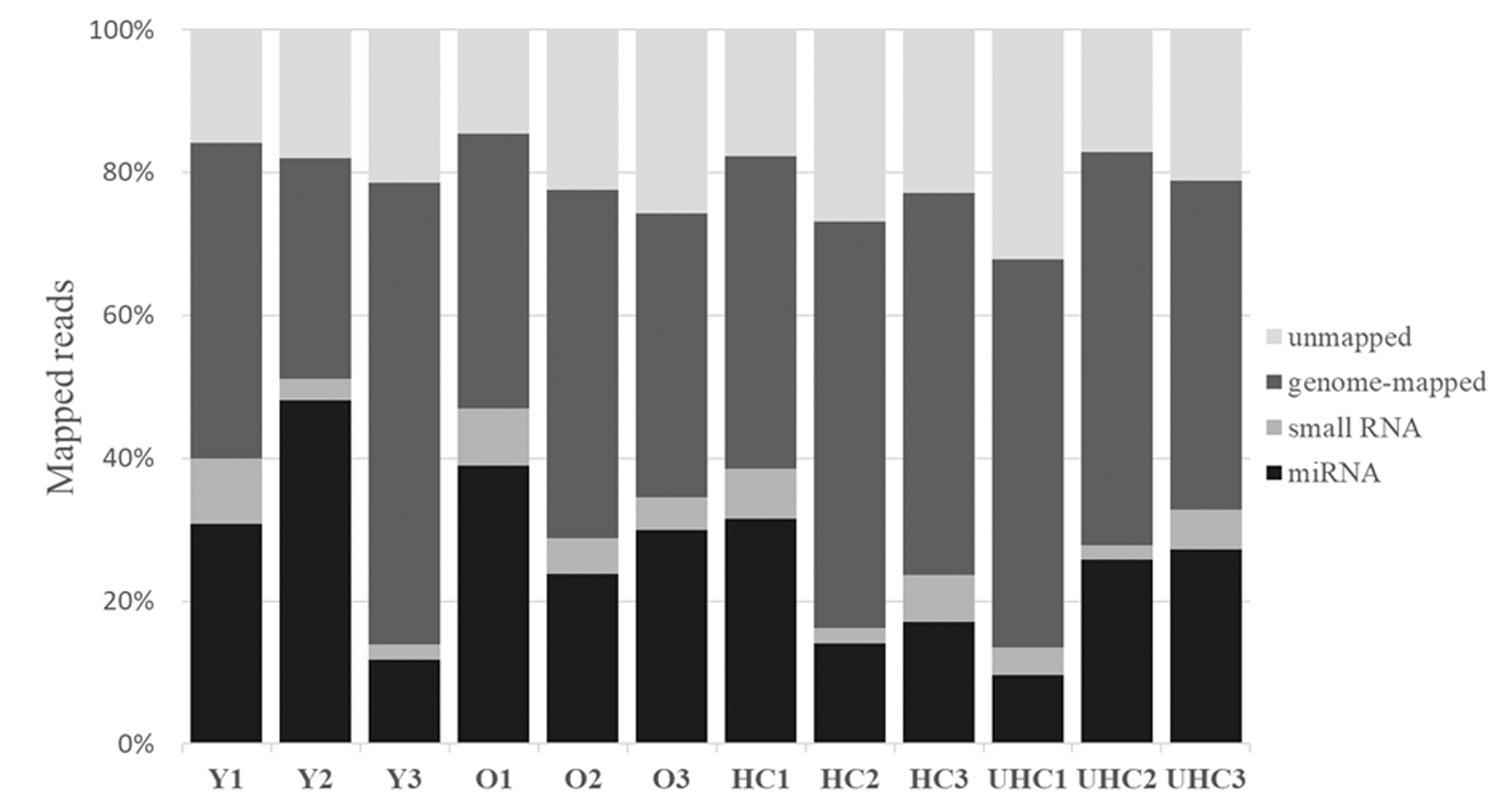

Supplement: Supplementary file 1 — Fig S1 [file ACEL-20-e13409-s002.tif]

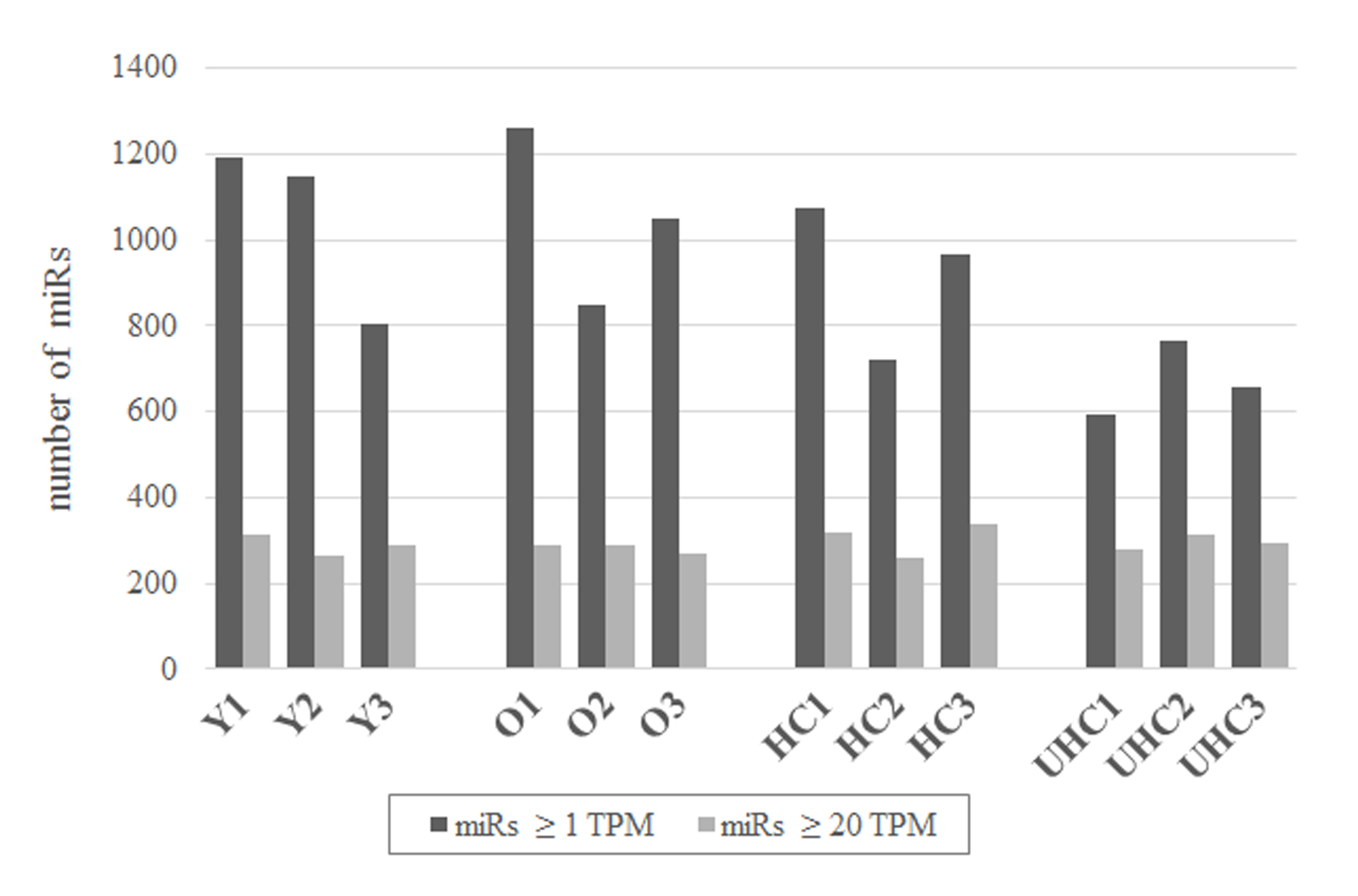

Supplement: Supplementary file 2 — Fig S2 [file ACEL-20-e13409-s003.tif]

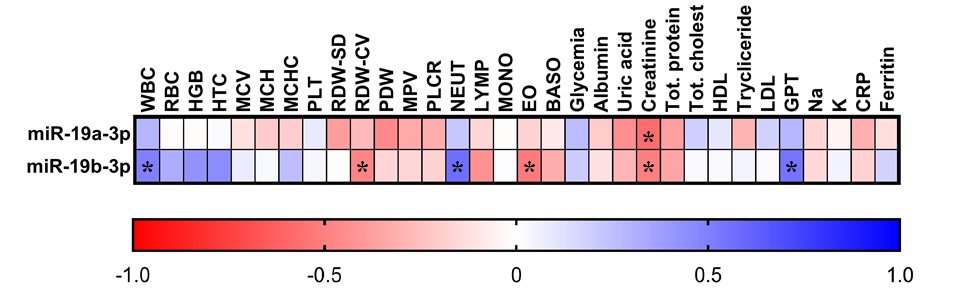

Supplement: Supplementary file 3 — Fig S3 [file ACEL-20-e13409-s004.tif]

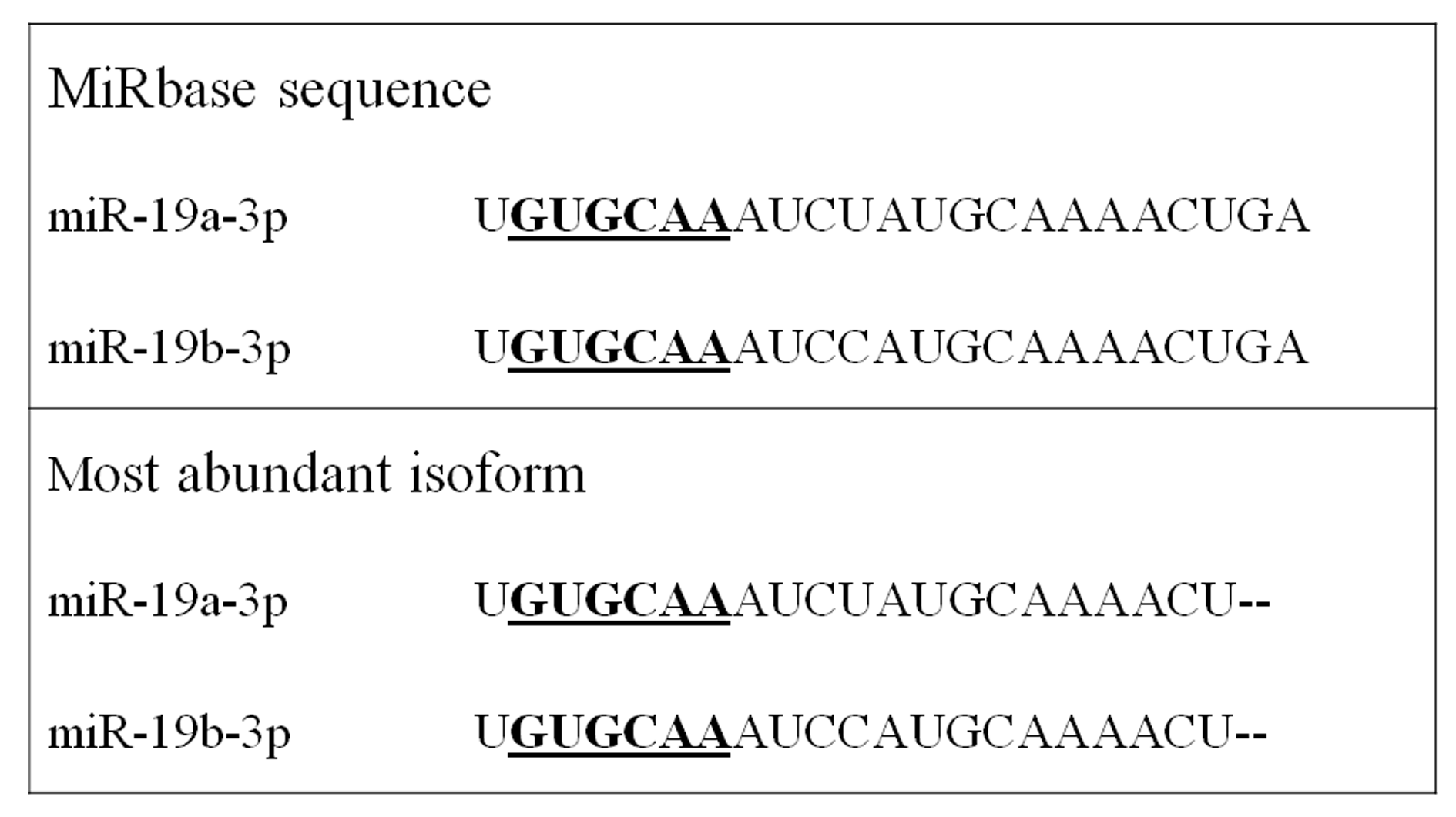

Supplement: Supplementary file 4 — Fig S4 [file ACEL-20-e13409-s005.tif]
