# Supplementary material for: Circulating miR‐19a‐3p and miR‐19b‐3p characterize the human aging process and their isomiRs associate with healthy status at extreme ages
Source: Aging Cell. 2021 Jun 23;20(7):e13409. doi: 10.1111/acel.13409 (PMC8282272; doi:10.1111/acel.13409)
Supplement: Supplementary file 5 — Supplementary Material [file ACEL-20-e13409-s001.docx]

**Table S1**. Total and aligned reads obtained from the sequencing

|  | **Total reads** | **Aligned reads** | **Precursor miR reads** | **Mature miR reads** |
| --- | --- | --- | --- | --- |
| **Y1** | 1.56E+07 | 6.04E+06 | 4.46E+03 | 5.10E+06 |
| **Y2** | 1.18E+07 | 6.17E+06 | 3.18E+03 | 5.78E+06 |
| **Y3** | 7.67E+06 | 1.30E+06 | 1.62E+03 | 9.26E+05 |
| **O1** | 2.10E+07 | 9.43E+06 | 5.74E+03 | 8.36E+06 |
| **O2** | 1.12E+07 | 3.40E+06 | 3.22E+03 | 2.76E+06 |
| **O3** | 1.45E+07 | 5.65E+06 | 8.98E+03 | 4.48E+06 |
| **HC1** | 6.97E+06 | 2.74E+06 | 1.86E+03 | 2.34E+06 |
| **HC2** | 3.90E+06 | 1.25E+06 | 8.58E+02 | 1.03E+06 |
| **HC3** | 4.10E+06 | 1.49E+06 | 1.08E+03 | 1.23E+06 |
| **UHC1** | 5.11E+06 | 8.26E+05 | 3.83E+02 | 6.38E+05 |
| **UHC2** | 4.51E+06 | 9.95E+05 | 7.37E+02 | 7.76E+05 |
| **UHC3** | 4.64E+06 | 7.25E+05 | 1.20E+03 | 4.60E+05 |

Y: young, O: old, HC: healthy centenarians, UHC: unhealthy centenarians

**Table S2**. TPM value for each group of study, reported as TPM mean (standard error)

| **Mature miRNA** | **Y** | **O** | **HC** | **UHC** |
| --- | --- | --- | --- | --- |
| hsa-miR-19b-3p | 346.5 (91.2) | 455.7 (88.9) | 48.2 (22.9) | 256.9 (44.3) |
| hsa-miR-19a-3p | 98.1 (21.7) | 144.7 (24.3) | 18 (7.3) | 112.1 (21.5) |
| hsa-miR-4433b-3p | 39.9 (23.4) | 41.9 (18) | 39.1 (12.3) | 202.6 (124.9) |
| hsa-miR-145-5p | 196.6 (23.4) | 251.7 (14.1) | 116.4 (24.5) | 417.9 (137.2) |
| hsa-miR-10b-5p | 1048.5 (167.3) | 1255.5 (142.4) | 616.2 (130.6) | 2115.8 (613.4) |
| hsa-miR-6503-3p | 3.2 (0.5) | 2.6 (1) | 0.3 (0.2) | 6.5 (5.3) |
| hsa-miR-4485-3p | 3 (1.1) | 16.3 (7.2) | 51.3 (12.1) | 12.8 (2) |
| hsa-miR-887-3p | 4.7 (1.2) | 5.9 (3.5) | 2.3 (0.6) | 14.5 (6.1) |
| hsa-miR-598-3p | 29.7 (1.1) | 20.8 (3.3) | 39.7 (7.4) | 9.2 (2.7) |
| hsa-miR-296-5p | 6.6 (2) | 5.6 (2.9) | 2.5 (1.3) | 13.7 (5.6) |
| hsa-miR-2277-5p | 1.9 (0.6) | 0.6 (0.5) | 4.6 (1.4) | 0 (0) |
| hsa-miR-6842-5p | 2 (0.9) | 0.2 (0.1) | 4.6 (1.7) | 0 (0) |
| hsa-miR-4739 | 0.4 (0.3) | 0 (0) | 0 (0) | 3.6 (3) |
| hsa-miR-122-5p | 1526.2 (133.1) | 2885.8 (306.4) | 1825.4 (523.4) | 649.4 (118.3) |
| hsa-miR-31-5p | 4.1 (0.8) | 12.4 (8.5) | 2.8 (0.9) | 0 (0) |
| hsa-miR-409-3p | 853.8 (201.2) | 412.4 (101.3) | 1753.7 (864.7) | 624.9 (230.2) |
| hsa-miR-3688-3p | 7 (2) | 4.8 (0.7) | 2.4 (0.6) | 0 (0) |
| hsa-miR-4741 | 0.1 (0.1) | 0 (0) | 0.6 (0.2) | 6.6 (2.6) |
| hsa-miR-197-5p | 1.2 (0.1) | 4.5 (2) | 2.8 (1.2) | 12.5 (2.7) |
| hsa-miR-4656 | 0 (0) | 0 (0) | 0 (0) | 2.2 (1.8) |
| hsa-miR-539-5p | 12.3 (4.2) | 6.3 (1.7) | 42.1 (18.6) | 12.5 (7.2) |
| hsa-miR-4669 | 2.9 (2.3) | 5.5 (0.8) | 2.4 (1.1) | 0 (0) |
| hsa-miR-487b-3p | 59.9 (14.1) | 33.1 (8.4) | 162.1 (71.5) | 59.9 (26.6) |
| hsa-miR-32-5p | 50.8 (20) | 23.7 (0.4) | 23.4 (5.9) | 9.1 (2.9) |
| hsa-miR-3168 | 1.6 (0.2) | 3.7 (2.7) | 6.6 (5) | 17.5 (7.6) |
| hsa-miR-127-3p | 650 (220.5) | 398.1 (125.3) | 1512.6 (564.5) | 657.2 (219.5) |
| hsa-miR-3200-3p | 29.9 (6.3) | 39.2 (11.1) | 21.1 (2.7) | 7.7 (3.6) |
| hsa-miR-1226-5p | 0 (0) | 0 (0) | 0.3 (0.2) | 3.8 (2.1) |
| hsa-miR-431-5p | 99.2 (23) | 51.1 (13.5) | 231.2 (98.1) | 92.4 (29.7) |
| hsa-miR-3176 | 9.6 (2.6) | 6.5 (1.2) | 5.8 (2.1) | 1.2 (0.5) |
| hsa-miR-96-5p | 7.3 (2) | 6.7 (1.3) | 1.2 (0.7) | 4.6 (3.3) |
| hsa-miR-323b-3p | 84.2 (27.3) | 40 (11.6) | 214.9 (79.1) | 81.6 (39.1) |
| hsa-miR-134-5p | 211 (80.6) | 134.2 (23.1) | 790.3 (352.4) | 363 (175.8) |
| hsa-miR-6817-3p | 0.1 (0.1) | 0 (0) | 0.4 (0.2) | 3.7 (1.1) |
| hsa-miR-3675-5p | 0 (0) | 0.1 (0.1) | 1.8 (0.4) | 0 (0) |
| hsa-miR-101-5p | 23.6 (3.1) | 25 (9.2) | 8 (2.7) | 2.6 (1.6) |
| hsa-miR-215-5p | 17.2 (4.3) | 28 (4.2) | 10.3 (4.9) | 21.5 (6.8) |
| hsa-miR-6087 | 46.1 (16.3) | 69.2 (19.7) | 26.3 (3.6) | 61.2 (30.1) |
| hsa-miR-379-5p | 74.9 (18.5) | 56.5 (19.4) | 331.3 (124.6) | 169.7 (106.7) |
| hsa-miR-186-3p | 18.3 (3.3) | 20.4 (2.9) | 4.7 (2.1) | 13.3 (3.2) |
| hsa-miR-144-3p | 3581.3 (541.3) | 3943.6 (595.9) | 2683.6 (716) | 1544.6 (446.2) |
| hsa-miR-362-5p | 3.1 (1) | 3.4 (0.7) | 1 (0.5) | 0 (0) |
| hsa-miR-382-5p | 243.4 (78.2) | 198 (56.6) | 1000.1 (406.6) | 507.4 (240.8) |
| hsa-miR-93-5p | 4759.6 (769) | 3956.8 (327.3) | 3763.7 (1079.5) | 2077 (602.2) |
| hsa-miR-576-5p | 23.3 (6) | 16.2 (1.2) | 12.6 (1.6) | 5.9 (0.5) |
| hsa-miR-654-5p | 30.3 (8.3) | 27.5 (7.4) | 129.4 (45.2) | 65.2 (30) |
| hsa-miR-186-5p | 2337 (244.1) | 2443.6 (112.9) | 978.1 (203.3) | 1592.3 (196.9) |
| hsa-miR-193b-3p | 3.5 (0.6) | 3.9 (2.8) | 0.8 (0.4) | 0 (0) |
| hsa-miR-493-5p | 102.1 (16.5) | 71.4 (24.9) | 303.8 (100.6) | 182.5 (111.2) |
| hsa-miR-183-5p | 1031 (145.5) | 1163.4 (163.4) | 404.3 (72.1) | 718.8 (252.6) |
| hsa-miR-9-5p | 6.5 (2.7) | 3.5 (1.4) | 10.3 (3.8) | 21.2 (4.6) |
| hsa-miR-4532 | 449.2 (200.8) | 483.2 (187.2) | 175.2 (71.9) | 243.1 (162) |
| hsa-miR-124-3p | 0.3 (0.1) | 0.1 (0) | 0.4 (0.1) | 1.4 (0.6) |
| hsa-miR-20b-5p | 163.2 (49.3) | 128.3 (10.5) | 80.4 (20.2) | 48.8 (14.1) |
| hsa-miR-144-5p | 230.5 (56.2) | 137.3 (17.2) | 98.8 (22.8) | 56.9 (4.6) |
| hsa-miR-18b-5p | 8.6 (2.7) | 8 (2) | 1.9 (0.8) | 4.7 (2.6) |
| hsa-miR-29c-3p | 114.6 (8.8) | 131.7 (28.2) | 48.5 (4.5) | 77.4 (30.4) |
| hsa-miR-1908-3p | 1.5 (0.3) | 3.7 (1.4) | 7.5 (1.4) | 14.4 (6.1) |
| hsa-miR-16-5p | 54563.2 (21378.3) | 32208.3 (4074.1) | 14675.6 (2272.7) | 20901.1 (6423.8) |
| hsa-miR-3135b | 1.9 (1.2) | 5.8 (1.7) | 13.1 (1.8) | 8.4 (1) |
| hsa-miR-1299 | 33.1 (23.6) | 6.6 (3) | 8.9 (5.5) | 5.4 (3.6) |
| hsa-miR-432-5p | 517.2 (139.8) | 391.8 (146.2) | 1510.6 (517.5) | 1131.6 (519) |
| hsa-miR-4488 | 16.3 (7.6) | 35.9 (11.5) | 83.5 (45.4) | 78.7 (29.7) |
| hsa-miR-550a-3p | 22.6 (3.4) | 15.9 (2.6) | 7.1 (1.5) | 5.5 (0.9) |
| hsa-miR-106b-5p | 472.7 (121.3) | 396.6 (111.5) | 172.6 (27.6) | 125.1 (22.2) |
| hsa-miR-15b-5p | 269.4 (46.7) | 155 (13.7) | 129.9 (5.7) | 106.8 (19.8) |
| hsa-miR-194-5p | 59.8 (10.5) | 39.7 (7.7) | 25.1 (6.1) | 17.2 (4.5) |
| hsa-miR-4466 | 1.9 (0.3) | 5.7 (0.4) | 12.4 (4) | 17.5 (10.7) |
| hsa-miR-323a-3p | 51.1 (3.3) | 31.9 (10.1) | 155.8 (59.8) | 107.2 (41) |
| hsa-miR-5189-3p | 0.1 (0) | 2.3 (1) | 0.6 (0.2) | 1.2 (0.5) |
| hsa-miR-490-5p | 2.1 (0.8) | 2.9 (0.5) | 17.2 (8.3) | 10.7 (7.2) |
| hsa-miR-3138 | 7.4 (2.2) | 12 (2.8) | 30.3 (9) | 35.2 (8.4) |
| hsa-miR-192-5p | 1198.4 (72.8) | 1341.5 (164.4) | 524.5 (14.9) | 575.8 (109.2) |
| hsa-miR-185-5p | 2037.2 (394.5) | 2213.8 (704.5) | 849.5 (125.3) | 755.3 (113.2) |
| hsa-miR-3200-5p | 29.4 (4) | 24.9 (2.7) | 6.9 (2.3) | 5.9 (1.9) |
| hsa-miR-15a-5p | 3248.8 (846) | 1955.7 (333.9) | 1399.6 (106.5) | 1318.8 (502.6) |
| hsa-miR-451a | 95693.9 (23647.7) | 58829.1 (18865.2) | 20108.4 (792.3) | 18741.8 (5151.7) |
| hsa-miR-3161 | 2.2 (0.9) | 0.4 (0.3) | 0 (0) | 0 (0) |
| hsa-miR-217 | 0 (0) | 1.6 (0.3) | 0 (0) | 0 (0) |

Y: young, O: old, HC: healthy centenarians, UHC: unhealthy centenarians

**Table S3**. Hematobiochemical parameters collected from young (Y), old (O) and centenarians (C) analyzed in the validation phase

|  | **Y** (N=10-13) | **O** (N=2-15) | **C** (N=13-16) |
| --- | --- | --- | --- |
|  | Mean ± sd | Mean ± sd | Mean ± sd |
| White blood cells (x10^9^/L) | 5.6 ± 0.8 | 5.4 ± 1.5 | 6.4 ± 1.7 |
| Red blood cells (x10^12^/L) | 4.9 ± 0.4 | 4.9 ± 0.4 | 3.8 ± 0.6 ^# †^ |
| Hemoglobin (g/dL) | 14.5 ± 1.1 | 14.1 ± 1.3 | 11.1 ± 1.9 ^# †^ |
| Hematocrit (%) | 44 ± 2.6 | 42.9 ± 3.7 | 35.5 ± 5.5 ^# †^ |
| Mean cell volume (fL) | 89.2 ± 2.6 | 88.1 ± 7.1 | 93.5 ± 8.2 |
| Mean cell hemoglobin (pG) | 29.4 ± 1.1 | 29 ± 2.7 | 29.4 ± 2.5 |
| Mean cell hemoglobin concentration (g/dL) | 32.9 ± 1 | 32.9 ± 1.1 | 31.4 ± 1.1 ^# †^ |
| Red blood cells distribution width - coefficient of variation (%) | 12.9 ± 0.6 | 13.4 ± 0.8 | 15.4 ± 1.7 ^# †^ |
| Red blood cells distribution width - standard deviation (fL) | 41.3 ± 1.8 | 44.9 ± 1.8 | 50.3 ± 9.6 ^#^ |
| Platelet count (x10^9^/L) | 237.2 ± 33.4 | 238.5 ± 61 | 238.6 ± 69.7 |
| Platelet distribution width (%) | 13.4 ± 2 | 33.9 ± 21.2 | 20.3 ± 15.7 |
| Mean platelet volume (fL) | 10.7 ± 0.8 | 9.5 ± 1.3 ^◊^ | 10.5 ± 1.2 |
| Platelet large cell ratio (fL) | 30.7 ± 5.7 | 27.6 ± 4.2 | 29.9 ± 8.9 |
| Neutrophils (x10^9^/L) | 53 ± 6.5 | 37.6 ± 24.5 | 56.1 ± 8.1 |
| Lymphocytes (x10^9^/L) | 34.3 ± 5.9 | 23 ± 15.3 | 30.8 ± 7 |
| Monocytes (x10^9^/L) | 8.7 ± 1.2 | 4.3 ± 3.1 ^◊^ | 8.5 ± 2.8 ^†^ |
| Eosinophils (x10^9^/L) | 3.3 ± 2.3 | 2.2 ± 1.7 | 4.1 ± 3.3 |
| Basophils (x10^9^/L) | 0.7 ± 0.8 | 0.4 ± 0.4 | 0.4 ± 0.3 |
| Glycemia (mg/dL) | 84.8 ± 4.6 | 84.5 ± 16.8 | 87.1 ± 8.5 |
| Albumin (g/dL) | 4.6 ± 0.2 | 4.3 ± 0.2 | 3.6 ± 0.5 ^# †^ |
| Uric acid (mg/dL) | 4.5 ± 0.7 | 5.4 ± 1.1 | 6.2 ± 2.3 |
| Creatinine (mg/dL) | 0.9 ± 0.1 | 1 ± 0.2 | 1.5 ± 0.8 ^# †^ |
| Total protein (g/dL) | 7.2 ± 0.4 | 7.2 ± 0.1 | 6.9 ± 0.5 |
| Total cholesterol (mg/dL) | 187.1 ± 24.5 | 206.9 ± 43.3 | 199.3 ± 37.8 |
| HDL cholesterol (mg/dL) | 57 ± 13.9 | 59.9 ± 12.6 | 58.9 ± 17.5 |
| Triglycerides (mg/dL) | 76.4 ± 30.1 | 158.4 ± 120.2 ^◊^ | 104.6 ± 24.9 ^#^ |
| LDL cholesterol (mg/dL) | 118.5 ± 18.3 | 125.5 ± 0.5 | 119.6 ± 33.3 |
| GPT transaminase (U/L) | 16.5 ± 8.8 | 16.2 ± 4.9 | 7.3 ± 2.6 ^# †^ |
| Sodium (mmol/L) | 140.6 ± 1.8 | 141.2 ± 1.2 | 142.1 ± 4.7 |
| Potassium (mmol/L) | 4.3 ± 0.3 | 4.5 ± 0.3 | 4.5 ± 0.6 |
| C-protein reactive (mg/L) | 2.1 ± 2.6 | 0.9 ± 1.8 ^◊^ | 6.5 ± 7.7 ^†^ |
| Ferritin (ng/mL) | 63.2 ± 51.9 | 46.4 ± 5.1 | 112.9 ± 136.7 |

Significant variations (p<0.05): ◊ O vs Y; # C vs Y; † C vs Y

**Table S4**. Hematobiochemical parameters collected from centenarians analyzed in the validation phase, 11 healthy (HC) and 6 unhealthy centenarians (UHC)

|  | **HC** (N=8-11) | **UHC** (N=5-6) |  |
| --- | --- | --- | --- |
|  | Mean ± sd | Mean ± sd | p value |
| White blood cells (x10^9^/L) | 5.7 ± 0.9 | 7.5 ± 2.2 | ns |
| Red blood cells (x10^12^/L) | 3.9 ± 0.7 | 3.6 ± 0.3 | ns |
| Hemoglobin (g/dL) | 11.5 ± 1.8 | 10.1 ± 1.5 | ns |
| Hematocrit (%) | 36.2 ± 5.4 | 33.0 ± 4.6 | ns |
| Mean cell volume (fL) | 94.1 ± 7.3 | 92.6 ± 9.9 | ns |
| Mean cell hemoglobin (pG) | 29.9 ± 1.9 | 28.4 ± 3.3 | ns |
| Mean cell hemoglobin concentration (g/dL) | 31.8 ± 0.7 | 30.4 ± 1.2 | 0.039 |
| Red blood cells distribution width - coefficient of variation (%) | 14.7 ± 0.9 | 16.3 ± 2.0 | ns |
| Red blood cells distribution width - standard deviation (fL) | 49.5 ± 4.4 | 50.3 ± 14.9 | ns |
| Platelet count (x10^9^/L) | 212.3 ± 48.4 | 267.0 ± 96.4 | ns |
| Platelet distribution width (%) | 13.7 ± 2.7 | 12.6 ± 3.7 | ns |
| Mean platelet volume (fL) | 10.8 ± 1.1 | 10.4 ± 1.6 | ns |
| Platelet large cell ratio (fL) | 31.1 ± 7.3 | 28.3 ± 11.5 | ns |
| Neutrophils (x10^9^/L) | 54.5 ± 9.2 | 57.6 ± 5.5 | ns |
| Lymphocytes (x10^9^/L) | 32.2 ± 8.5 | 29.4 ± 2.4 | ns |
| Monocytes (x10^9^/L) | 8.1 ± 2.6 | 8.7 ± 3.1 | ns |
| Eosinophils (x10^9^/L) | 4.8 ± 3.8 | 3.5 ± 2.3 | ns |
| Basophils (x10^9^/L) | 0.5 ± 0.3 | 0.4 ± 0.2 | ns |
| Glycemia (mg/dL) | 88.7 ± 8.8 | 84.3 ± 7.0 | ns |
| Albumin (g/dL) | 3.8 ± 0.5 | 3.3 ± 0.2 | 0.023 |
| Uric acid (mg/dL) | 7.0±2.4 | 4.8 ± 1.4 | ns |
| Creatinine (mg/dL) | 1.7±0.9 | 1.1 ± 0.1 | ns |
| Total protein (g/dL) | 7.1±0.5 | 6.5 ± 0.3 | 0.046 |
| Total cholesterol (mg/dL) | 203.2 ± 36.0 | 192.8 ± 40.0 | ns |
| HDL cholesterol (mg/dL) | 65.1 ± 18.1 | 48.5 ± 9.8 | ns |
| Triglycerides (mg/dL) | 102.2 ± 25.1 | 108.7 ± 24.1 | ns |
| LDL cholesterol (mg/dL) | 117.7 ± 32.4 | 122.8 ± 34.5 | ns |
| GPT transaminase (U/L) | 6.7 ± 2.1 | 8.3 ± 2.9 | ns |
| Sodium (mmol/L) | 140.8 ± 2.3 | 144.2 ± 6.6 | ns |
| Potassium (mmol/L) | 4.4 ± 0.6 | 4.7 ± 0.4 | ns |
| C-protein reactive (mg/L) | 3.4 ± 4.2 | 11.6 ± 9.3 | ns |
| Ferritin (ng/mL) | 111.0 ± 149.3 | 116.1 ± 113.4 | ns |

**Table S5**. Significant pathways in common between miR-19a-3p and miR-19b-3p

| **KEGG pathway** | **p-value** |
| --- | --- |
| Proteoglycans in cancer | <1e-325 |
| Viral carcinogenesis | 2.41E-11 |
| Prolactin signaling pathway | 9.48E-09 |
| Glioma | 1.88E-08 |
| FoxO signaling pathway | 3.19E-07 |
| p53 signaling pathway | 7.57E-07 |
| Prostate cancer | 1.14E-06 |
| Melanoma | 2.04E-06 |
| ECM-receptor interaction | 3.84E-06 |
| Signaling pathways regulating pluripotency of stem cells | 4.08E-06 |
| Non-small cell lung cancer | 7.28E-06 |
| Sphingolipid signaling pathway | 8.25E-06 |
| Estrogen signaling pathway | 3.78E-05 |
| Chronic myeloid leukemia | 4.06E-05 |
| Hepatitis B | 4.72E-05 |
| Central carbon metabolism in cancer | 6.65E-05 |
| Colorectal cancer | 0.00013 |
| Adrenergic signaling in cardiomyocytes | 0.00018 |
| Endometrial cancer | 0.00032 |
| Progesterone-mediated oocyte maturation | 0.00034 |
| Thyroid hormone signaling pathway | 0.00035 |
| AMPK signaling pathway | 0.00039 |
| Pancreatic cancer | 0.00052 |
| Type II diabetes mellitus | 0.00094 |
| Bladder cancer | 0.00172 |
| Transcriptional misregulation in cancer | 0.00175 |
| TGF-beta signaling pathway | 0.00480 |
| PI3K-Akt signaling pathway | 0.00861 |

**FIGURE LEGENDS**

**Figure S1. Mapped reads for each sample.** Summary of the mapped reads to human genome are here reported as percentage for each sample. Unmapped reads are reads not aligned to the reference genome. Genome-mapped are reads aligned to the reference genome, except small RNA and miR sequences. Small RNA are reads aligned to small RNA database except miR sequences which are considered apart in this analysis. MiRNA are reads aligned to miRbase database. Results were elaborated by Exiqon A/S company (Vedbæk, Denmark).

**Figure S2.** **Number of identified miRs for each sample after normalization.** Number of miRs identified by the sequencing are shown. MiRs were sequenced using plasma samples obtained from 3 young donors (Y1, Y2, Y3), 3 old donors (O1, O2, O3), 3 healthy centenarians (HC1, HC2, HC3) and 3 unhealthy centenarians (UHC1, UHC2, UHC3). Data were normalized with TPM method (Tags Per Million) setting a minimum count threshold as 20 TPM for subsequent analyses.

**Figure S3.** **Spearman correlation matrix in centenarians.** RT-qPCR miR-19a/b-3p levels were analyzed with Spearman test and 7 significant correlations were identified (*, p ≤ 0.05). Matrix correlation is here reported, showing different color gradients according to Spearman R values. WBC white blood cells, RBC red blood cells, HGB hemoglobin, HTC hematocrit, MCV mean cell volume, MCH mean cell hemoglobin, MCHC mean cell hemoglobin concentration, PLT platelet count, RDW-SD red blood cells distribution width - standard deviation, RDW-CV red blood cells distribution width - coefficient of variation, PDW platelet distribution width, MPV mean platelet volume, PLCR platelet large cell ratio, NEUT neutrophils, LYMPH lymphocytes, MONO monocytes, EO eosinophils, BASO basophils, HDL high-density lipoprotein, LDL low-density lipoprotein, GPT alanine aminotransferase, Na sodium, K potassium, CRP c-protein reactive.

**Figure S4. miR-19a/b-3p base sequences and their most abundant 3’ isomiRs.** The annotated sequences in miRBase and the most abundant isoforms revealed by small RNA-seq are reported for miR-19a/b-3p. The two miRs share the same seed region (underlined and in bold) and the most abundant isomiRs are a truncated form at 3’.
